# Intranasal dexmedetomidine: does the delivery device matter?^☆^

**DOI:** 10.1016/j.bjane.2026.844791

**Published:** 2026-07-08

**Authors:** Eliane C.S. Soares, Paulo C.P. Figueiredo, Fabiano Soares Carneiro, Eduardo M.O. Melo e Silva, Patrícia B. Amaral

**Affiliations:** a Grupo SAM / Rede Mater Dei de Saúde, Belo Horizonte, MG, Brazil; b CET Hospital Universitário Ciências Médicas, Belo Horizonte, MG, Brazil; c Faculdade Ciências Médicas de Minas Gerais, Belo Horizonte, MG, Brazil

Dear Editor,

We read with great interest the systematic review and meta-analysis by Rocha et al. on intranasal dexmedetomidine (IN-DEX) for procedural sedation in children.^1^ This rigorous synthesis, encompassing 28 randomized controlled trials and more than 1,600 participants, provides a comprehensive quantitative framework for the clinical planning of IN-DEX monotherapy. The finding that doses of 2–3 µg.kg^-1^ yield an 84% success rate with high-quality evidence represents a meaningful advance for pediatric sedation practice.

Beyond these contributions, we wish to highlight an important variable not addressed in the analysis, namely the device used for intranasal delivery. In clinical practice, there is a widespread assumption that effective IN-DEX administration requires a mucosal atomization device, which disperses the drug as a fine mist (particle size 30–100 µm) to maximize mucosal contact and absorption.^2^ Simple instillation via a standard 1 mL syringe (the “drops” technique) is often regarded as an inferior alternative, despite being universally available and requiring no additional equipment or cost. Notably, among the trials that specified the delivery device (15 of 28), drops were the most frequently reported method, applied in 10 studies.

Two trials directly compared both delivery methods.^2^^,^^3^ Li et al. (2016), included in the meta-analysis, represents the largest prospective RCT to date directly comparing atomizer with drops (n = 279, children aged 2–36 months undergoing echocardiography, 3 µg.kg^-1^).^2^ The study found no statistically significant differences in sedation success or onset time between the two delivery methods in this specific population and clinical context; however, it was not designed or analyzed as a non-inferiority trial.^2^ Xie et al., not included in this meta-analysis, possibly due to differences in outcome definitions, similarly found that onset time and patient acceptance of intranasal administration were comparable between devices (23 vs. 25 min, p = 0.281; p = 0.242, respectively).^3^

These clinical observations align with pharmacokinetic data. The transmucosal bioavailability of dexmedetomidine has been estimated at 40.6% (95% CI 34.7%–54.4%) with atomization and 40.7% (95% CI 36.5%–53.2%) with drops (virtually identical values), reflecting the drug’s high lipophilicity and the nasal mucosa’s rich vascularity.^4^ Atomization may offer marginally faster onset than drops, yet this advantage does not consistently translate into a clinically meaningful difference in sedation outcomes, particularly when the drug is administered in the small volumes permitted by dexmedetomidine’s commercial concentration of 100 µg.mL^-1^.

Beyond pharmacology, this question carries direct implications for healthcare access. Drop administration requires no specialized equipment beyond a standard 1 mL syringe, which is universally available in any clinical setting. As reported by Li et al.,^2^ the institutional cost of atomizer use has been estimated at approximately USD 13 per patient, while simple drops carry no additional cost. The recurring expenditure may discourage adoption even in well-resourced centers and may represent a meaningful barrier in low- and middle-income countries and in resource-limited emergency and primary care settings, where non-invasive, needle-free sedation would offer the greatest benefit.

Nevertheless, device choice may still matter in specific clinical contexts. In uncooperative or crying children, drops may be less reliable, as swallowing during administration reduces mucosal bioavailability, a limitation that the atomization device may partially mitigate.^3^ Similarly, when larger volumes are required, drops carry a greater risk of oropharyngeal runoff and partial oral absorption, which is particularly relevant given dexmedetomidine's poor oral bioavailability.^4^^,^^5^ This concern is especially applicable in older or heavier children, for whom dividing the dose equally between both nostrils is advisable to minimize runoff and maximize mucosal surface area.^5^ Nasal obstruction and excessive secretions, frequently observed in children selected for upper airway surgeries, should also be considered, as they may impair absorption regardless of delivery method.^5^ Finally, operator familiarity with the chosen device, whether a standard syringe or a atomization device, may also influence clinical outcomes in practice, independently of pharmacological considerations.^4^

We acknowledge that, although drop-based administration was reported in 10 of the included studies, the available direct comparative evidence between drops and atomization devices remains limited to two clinical trials with different designs, populations, and outcome measures,^2^^,^^3^ only one of which was included in the Rocha et al. meta-analysis.^1^ Nevertheless, our group has accumulated considerable clinical experience with nasal drop-based administration as a routine alternative to pre-anesthetic sedation for the last 5 years, and we respectfully wish to draw attention to this evidence, as many institutions still do not consider IN-DEX a viable option due to the perceived need for a mucosal atomization device.

We congratulate Rocha et al. on their rigorous and clinically relevant contribution and hope this perspective will stimulate further research to optimize intranasal sedation strategies.

Sincerely,

## Authors’ contributions

Eliane C.S. Soares: Conception and design of the study; acquisition, analysis and interpretation of data; drafting the article.

Paulo C.P. Figueiredo: Conception and design of the study; revising the article critically for important intellectual content.

Fabiano Soares Carneiro: Conception and design of the study; revising the article critically for important intellectual content.

Eduardo M.O. Melo e Silva: Acquisition and analysis of data; revising the article critically for important intellectual content.

Patrícia B. Amaral: Acquisition and analysis of data; revising the article critically for important intellectual content.

## Generative AI and AI-assisted technologies

During the preparation of this work, the authors used Claude (Anthropic version 3.5 Sonnet) and Grammarly (Grammarly Inc. version 1.2) to improve the English writing structure. After using these tools, the authors reviewed and edited the content as needed and take full responsibility for the content of the published article.

## Data availability statement

No new data were created or analyzed in this study. Data sharing is not applicable to this article.

## Funding

This study did not receive any funding.

## Conflicts of interest

The authors declare no conflicts of interest.
